# Circulating antithyroid antibodies contribute to the decrease of glomerular filtration rate in lithium-treated patients: a longitudinal study

**DOI:** 10.1186/s40345-017-0114-4

**Published:** 2018-03-01

**Authors:** Alberto Bocchetta, Luca Ambrosiani, Gioia Baggiani, Claudia Pisanu, Caterina Chillotti, Raffaella Ardau, Fernanda Velluzzi, Doloretta Piras, Andrea Loviselli, Antonello Pani

**Affiliations:** 1 Section of Neuroscience and Clinical Pharmacology, Department of Biomedical Sciences, University of Cagliari, Via Ospedale 54, 09124 Cagliari, Italy; 2 Unit of Clinical Pharmacology, Cagliari University Hospital, Cagliari, Italy; 3 Department of Medical Sciences and Public Health, University of Cagliari, Cagliari, Italy; 4 Nephrology, Dialysis and Transplantation Unit, ‘Giuseppe Brotzu’ Hospital, Cagliari, Italy

**Keywords:** Lithium, Renal insufficiency, Chronic, Glomerular filtration rate, Thyroid microsomal antibodies, Anti-thyroglobulin

## Abstract

**Background:**

Concerns about the adverse effects of long-term treatment with lithium include reduced renal function. In the present study, we examined comorbidities which may be associated with chronic kidney disease in a cohort of patients treated with lithium for up to 41 years.

**Methods:**

We studied 394 patients who were treated with lithium for ≥ 5 years. The potential role of comorbidities (diabetes, concurrent antihypertensive medication, treatment with l-thyroxine, and presence of antithyroid peroxidase/microsomes, anti-thyroglobulin, and/or anti-thyrotropin-receptor antibodies) was analysed. We focused on the categories of patients with an estimated glomerular filtration rate (eGFR) lower than 60 or 45 mL/min/1.73 m^2^ as calculated from serum creatinine according to the Modification of Diet in Renal Disease Study Group. We applied multivariate regression analysis and Cox survival analysis to study the effects exerted by sex, age, duration of lithium treatment, and comorbidities using eGFR categories as the dependent variable. Kaplan–Meier curves were generated to measure the time to decline to an eGFR lower than 45 mL/min/1.73 m^2^ in patients with positive or negative thyroid antibodies.

**Results:**

Age was associated with a decline to an eGFR lower than 60 mL/min/1.73 m^2^ after controlling for sex, duration of lithium treatment, and comorbidities. Circulating thyroid antibodies were associated with a decline to an eGFR lower than 45 mL/min/1.73 m^2^.

**Conclusions:**

The present study is the first to suggest a potential role of circulating thyroid antibodies in the severe decline of eGFR in lithium-treated patients.

## Background

Adverse renal effects of lithium have long been known, varying from very frequent reversible polyuria (Schou 1958) to irreversible kidney damage (Hestbech et al. 1977; Aurell et al. 1981). New studies have raised concern especially for patients treated for many years (Bendz et al. 2010) and the debate has been revived in 2012 by a review and meta-analysis of the lithium toxicity profile (McKnight et al. 2012). Thereafter, several studies based on various kinds of data sources have addressed the potential risk factors for chronic kidney disease (CKD) in lithium-treated patients. Principal factors under study include sex, age, duration of lithium treatment, concurrent medications, and comorbidities that are already known to predispose to CKD.

In an initial study of 139 patients from our lithium clinic, we found a positive correlation between the duration of lithium treatment and reduced estimated glomerular filtration rate (eGFR) (Bocchetta et al. 2013). Subsequently, we extended the study to 953 patients treated for at least one year and found that eGFR was lower in women, in older patients, and in patients with longer duration of lithium treatment (Bocchetta et al. 2015).

In the meantime, several studies began to be published exploring population-based health records, mostly European, to identify factors which are potentially associated with CKD in lithium-treated cohorts. According to three large studies based on thousands of subjects from UK and Denmark, it can be concluded that long-term exposure to lithium is associated with increased rates of CKD (Close et al. 2014; Kessing et al. 2015; Hayes et al. 2016). Diabetes and the use of antihypertensive diuretic drugs were found among potential risk factors for CKD (Close et al. 2014). On the contrary, a Scottish population-based cohort study including 305 patients exposed to lithium has come up with the different conclusion that there is no effect of stable lithium maintenance therapy on the rate of change in eGFR over time (Clos et al. 2015). However, it must be noted that the mean duration of exposure to lithium was only for 55 months. In the Scottish study, significant predictors for eGFR decline included age, baseline eGFR, comorbidities, co-prescriptions of nephrotoxic drugs, and episodes of lithium toxicity (Clos et al. 2015).

More recently, we took part in a survey from 12 collaborating international sites including 312 patients treated in the lithium clinics for at least 8 years (Tondo et al. 2017). Medical comorbidities were found among risk factors for low eGFR, but were not examined in the details.

### Aims of the study

As we are in possession of the detailed clinical data regarding patients on maintenance lithium treatment over the last four decades, the aims of this study were to determine whether comorbidities (hypertension, diabetes, thyroid function, and presence of circulating thyroid antibodies) or their treatments were associated with incident CKD.

## Methods

Clinical records of the patients in the database of the Unit of Clinical Pharmacology, Azienda Ospedaliero-Universitaria, Cagliari, were examined. Our unit has been one of the reference centres for lithium monitoring in the Cagliari area since its introduction in the 1970s.

Lithium monitoring was in line with the international guidelines. Our current therapeutic range for lithium maintenance is 0.50–0.80 mmol/L, after it had been lowered from the range 0.60–1.0 mmol/L which was used up to the 1990s (Baldessarini 1996).

The subjects included in this study were all the consecutive patients from the database who had been followed at the lithium clinic between 1976 and 2016 for at least 5 years and whose thyroid-antibody status was known.

For the purpose of this study, we extracted the following variables from the charts of the patients: demographic characteristics, age of onset of lithium treatment, duration of lithium treatment, concurrent antihypertensive medications, diagnosis of diabetes, data on renal and thyroid function.

### Renal function

Serum creatinine concentrations were taken from the panel of laboratory tests requested on an annual basis. We used the traditional standardisation method for serum creatinine. The estimated glomerular filtration rate (eGFR) was calculated from serum creatinine values using the equation proposed by the Modification of Diet in Renal Disease (MDRD) Study Group (Levey et al. 2009; Earley et al. 2012), with the ‘186’ correction factor, which takes age, sex and ethnicity into account. The following categories of eGFR were considered: higher than 90 mL/min/1.73 m^2^ (G1); 60–89 mL/min/1.73 m^2^ (G2); 45–59 mL/min/1.73 m^2^ (G3a); 30–44 mL/min/1.73 m^2^ (G3b); 15–29 mL/min/1.73 m^2^ (G4); lower than 15 mL/min/1.73 m^2^ (G5). The abbreviations and ranges recall those used by Kidney Disease Improving Global Outcomes (KDIGO) 2012 Clinical Practice Guidelines for the Evaluation and Management of Chronic Kidney Disease (CKD) (2012), but it must be noted that KDIGO CKD stages are also based on albuminuria categories, which were not included in the present study.

### Concomitant medical conditions and related treatments

We studied the following conditions potentially predisposing to CKD:

- Long-term treatment with any antihypertensive agent before declining to an eGFR lower than 60 mL/min/1.73 m^2^.
- Long-term treatment with oral hypoglycemic agents or insulin, or dietary treatment after receiving an established diagnosis of diabetes.
- Long-term treatment with l-thyroxine or antithyroid agents.
- Presence of one or more of the following circulating antibodies: anti-thyroid peroxidase (AbTPO), anti-thyroid microsomes (AbM), anti-thyroglobulin (AbTG), anti-thyrotropin receptors (TRAb). The laboratory tests varied over the decades. The antibodies were considered positive when they exceeded the upper end of the laboratory range in use at that time. For example, the cut-off was an AbM or AbTG titre ≥ 1:100 in the 1990s or an AbTPO ≥ 20 or ≥ 35 IU/mL (depending on the laboratory) in the more recent tests.

### Statistical analysis

We used Student’s *t* test to compare mean and Fisher’s exact test to analyse contingency tables. We applied multivariate regression analysis to study the effect exerted by demographic and clinical variables on renal function, using the eGFR category as the dependent variable. The independent variables which were analysed include: sex, age, duration of lithium treatment, diabetes, treatment with antihypertensive medication, treatment with l-thyroxine, and presence of thyroid antibodies. We used Cox survival analysis to study the effects exerted by sex, age, duration of lithium treatment, and comorbidities on renal function. Kaplan–Meier curves were generated to measure the time taken to enter stage G3b in the subgroups of the patients with positive or negative circulating thyroid antibodies. The Log-rank (Mantel–Cox) test was used to compare the survival distributions of the subgroups with positive or negative circulating thyroid antibodies.

Statistical analysis was performed using the SPSS statistical software package v20 (SPSS, Inc., USA).

## Results

Psychiatric diagnoses according to ICD-10 were the following: bipolar affective disorder (F31), *N* = 280 (bipolar I, 198; bipolar II, 82); schizoaffective disorder (F25), *N* = 33 (manic type, 20; depressive type, 13); recurrent depressive disorder (F33) *N* = 81.

Table 1 shows demographic and clinical characteristics of the 394 patients studied. Men had started lithium treatment earlier than women and were younger when studied. Duration of lithium treatment did not differ significantly between the sexes.

**Table 1:** Demographic and clinical characteristics by sex

	Total sample (*N* = 394)	Women (*N* = 289)	Men (*N* = 105)	*P* value
Age of onset of lithium treatment, mean ± SD	39.2 ± 13.0	40.7 ± 13.1	35.0 ± 11.8	0.0001
Years with lithium treatment, mean ± SD	15.1 ± 7.5	15.2 ± 7.7	14.7 ± 6.9	0.56
Current age, mean ± SD	55.0 ± 13.6	56.5 ± 13.6	50.8 ± 12.9	0.0002
Treatment with antihypertensive agents	132 (34%)	99 (34%)	33 (31%)	0.63
Diabetes	89 (23%)	63 (22%)	26 (25%)	0.59
Treatment with l-thyroxine	125 (32%)	114 (39%)	11 (10%)	0.0001
Circulating thyroid antibodies	102 (26%)	90 (31%)	12 (11%)	0.0001

Similar proportions of women and men were taking long-term antihypertensive drugs before declining to an eGFR lower than 60 mL/min/1.73 m^2^.

Diabetes was diagnosed in 89 (23%) patients: when last observed, 14 patients (9 women, 5 men) were being treated with insulin, 34 (21 women, 13 men) with oral hypoglycemic agents, and 41 (33 women, 8 men) with dietary treatment.

Only four patients (1%) had been treated with methimazole for hyperthyroidism, whereas treatment with L-thyroxine regarded as many as 125 (32%) patients. More women than men were taking treatment with l-thyroxine.

Prevalence of circulating thyroid antibodies was significantly higher in women. Antibodies were principally represented by AbTPO (or AbM in older tests prior to the identification of TPO as the specific thyroid microsomal antigen) alone or associated with AbTG (79% of cases). AbTG without AbTPO/AbM were found in 18 women and 1 man. We found only two cases of TRAb with no other circulating antibodies. However, it must be noted that TRAb is not in the panel of first instance thyroid function tests.

Antibody-positive women were taking l-thyroxine more frequently (54/90 = 60%) compared to the antibody-negative (60/199 = 30%) (*P* = 0.0001).

Table 2 shows demographic and clinical characteristics of patients by their final eGFR category. Overall, 84 patients had declined to an eGFR lower than 45 mL/min/1.73 m^2^ (CKD3b). A greater proportion of patients in the G3b subgroup had been treated with antihypertensive agents before the diagnosis of CKD compared to the G1–G2 subgroup.

**Table 2:** Demographic and clinical characteristics by the patient’s final eGFR category

	G3b (*N* = 84)	G3a (*N* = 55)	G1–G2 (*N* = 255)	Between-group differences (*P* value)
Number of males	13 (15%)^a^	18 (33%)	74 (29%)^b^	*a* < *b* (0.01)
Current age, mean ± SD	59 ± 11^c^	56 ± 13^d^	48 ± 21^e^	*c* > *e* (0.0001); *d* > *e* (0.007)
Treatment with antihypertensive agents before declining to eGFR < 60 mL/min/1.73 m^2^	38 (45%)^f^	22 (40%)	72 (28%)^g^	*f* > *g* (0.005)
Diabetes	21 (25%)	18 (33%)	50 (20%)	–
Treatment with l-thyroxine				
Males	4 (31%)^h^	2 (11%)	5 (7%)^i^	*h* > *i* (0.03)
Females	32 (45%)	14 (38%)	68 (38%)	
Total	36 (43%)^j^	16 (29%)	73 (29%)^k^	*l* > *m* (0.02)
Circulating thyroid antibodies				
Males	4 (31%)^l^	2 (11%)	6 (8%)^m^	*n* > o (0.04)
Females	29 (41%)	8 (22%)	53 (29%)	–
Total	33 (39%)^n^	10 (18%)	59 (23%)^o^	*p* > *q* (0.005)

Superscripts a–o are used to indicate significant between-group differences

G1–G2, eGFR ≥ 60 mL/min/1.73 m^2^

G3a, eGFR = 45–59 mL/min/1.73 m^2^

G3b, eGFR < 45 mL/min/1.73 m^2^

In the regression analysis using three different eGFR categories (G1/G2; G3a; G3b) as the dependent variable, age was the only independent variable with significant effects (Table 3). When the regression analysis was limited to the severest stage (G3b), the effect of circulating thyroid antibodies was the only significant variable (Table 4).

**Table 3:** Regression analysis using three different eGFR categories (G1/G2; G3a; G3b) as the dependent variable

Independent variables	*P* value	Coefficient beta (95% CI)
Sex	0.99	0.001 (− 0.21 to 0.21)
Age	0.009	0.007 (0.002 to 0.012)
Duration of lithium treatment	0.08	0.01 (− 0.001 to 0.022)
Treatment with antihypertensive drugs	0.15	0.13 (− 0.05 to 0.31)
Diabetes	0.89	0.01 (− 0.18 to 0.21)
Treatment with l-thyroxine	0.48	0.07 (− 0.12 to 0.25)
Circulating thyroid antibodies	0.10	0.16 (− 0.03 to 0.35)

**Table 4:** Regression analysis using two different eGFR categories (G1/G2/G3a; G3b) as the dependent variable

Independent variables	*P* value	Coefficient beta (95% CI)
Sex	0.55	− 0.03 (− 0.14 to 0.07)
Age	0.054	0.003 (− 5.3 to 0.005)
Duration of lithium treatment	0.38	0.003 (− 0.003 to 0.009)
Treatment with antihypertensive drugs	0.13	0.07 (− 0.02 to 0.16)
Diabetes	0.69	− 0.02 (− 0.12 to 0.08)
Treatment with l-thyroxine	0.39	0.04 (− 0.05 to 0.13)
Circulating thyroid antibodies	0.03	0.11 (0.01 to 0.20)

The presence of circulating antibodies was the only significant variable in the Log-rank test or Cox survival analysis (Table 5).

**Table 5:** Log-rank test and Cox regression analysis of the time taken to enter CKD3b

	Univariate (Log-rank test)	Multivariate (Cox regression analysis)		
	*P* value	Hazard ratio	95% CI	*P* value
Age^a^	0.21	0.98	0.96–1.0	0.19
Sex	0.21	0.66	0.35–1.25	0.20
Treatment with antihypertensive drugs	0.74	1.02	0.64–1.60	0.93
Circulating thyroid antibodies	0.05	1.57	1.00–2.49	0.05
Treatment with l-thyroxine	0.91	0.83	0.52–1.34	0.45
Diabetes	0.49	0.93	0.56–1.57	0.80

^a^In the univariate analysis, three categories of age (< 45 years; 45–65 years; > 65 years) were used

**Fig. 1:**
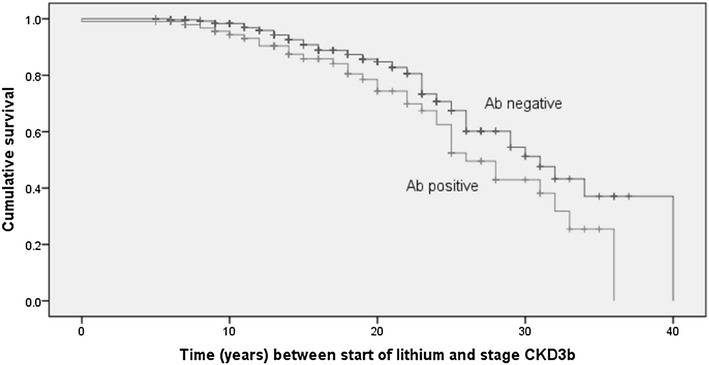
Kaplan–Meier curve showing the time taken between start of lithium treatment and the decline to stage CKD3b

Figure 1 shows the Kaplan–Meier curves measuring the time taken to enter stage CKD3b in the subgroups of patients positive (*N* = 102; 33 events; 32%) or negative (*N* = 292; 51 events; 17%) for circulating thyroid antibodies. In this case, the Log-rank (Mantel–Cox) test used to compare the survival distributions provided significant results (*P* = 0.044).

## Discussion

The problem of lithium-associated renal dysfunction has recently been addressed by several research studies and reviews. As already mentioned in the introduction, recent large studies agree that long-term exposure to lithium is associated with increased rates of CKD (Close et al. 2014; Bocchetta et al. 2015; Kessing et al. 2015; Hayes et al. 2016). Current interest has been devoted principally to risk factors that are already known to predispose to CKD. Results vary according to study setting, sample size, duration of lithium exposure, quality of data on confounders, and availability of a control group.

The purpose of the present study was to help clarify the role of concomitant conditions which may have been associated with CKD in our cohort of patients. From the beginning of lithium treatment, we have recorded any concurrent medication taken regularly in our clinical charts, as well as laboratory tests which are requested on an annual basis, and reports from consulting cardiologists, endocrinologists, and nephrologists. The main focus of the study was on antihypertensive treatment, diabetes and thyroid function with particular regard to thyroid antibodies. Although international guidelines do not generally include circulating thyroid antibodies among the routine laboratory tests for lithium patients, we have started recommending the search for antibodies since the late 1980s, when we first found evidence of high prevalence of AbM and/or AbTG in our cohort of patients (Bocchetta et al. 1991). Thereafter, as AbTPO had been identified as the specific thyroid microsomal antigen (Mariotti et al. 1987), we switched from AbM to AbTPO in our series of follow-up studies (for a review, see Bocchetta and Loviselli 2006).

### Diabetes and hypertension

In the present study, we were not able to reveal the role of diabetes and hypertension, that are already known to predispose to CKD in the general population (Stenvinkel 2010) and have also been associated with renal failure in patients treated with lithium (Close et al. 2014; Clos et al. 2015). It must be noted that we found only 14 cases of type-I diabetes, whereas cases of type-II diabetes were typically found in older patients and their effect was perhaps included in the age-related decline of eGFR that we already found in the entire cohort of patients treated with lithium (Bocchetta et al. 2015). A similar conclusion can also be made regarding the treatment with antihypertensive agents, whose apparent role (as shown in Table 2) vanished after controlling for age in the regression analysis. Similar results were observed in the UK study of the patients treated with lithium, (Close et al. 2014) that reported that hypertension itself was not associated with renal disease in fully adjusted models. Moreover, hypertension may not only predispose to CKD, but may also be a consequence of CKD (Sternlicht and Bakris 2017). It must also be noted that certain agents (namely ACE inhibitors and sartans) are prescribed to CKD patients even in the absence of hypertension to prevent further decline in eGFR (Ruggenenti et al. 2012), and this might influence results obtained by studies using the database of antihypertensive prescriptions as a proxy of a diagnosis of hypertension.

### Thyroid function

In the present study, we did not observe cases of clinical hypothyroidism or hyperthyroidism because patients were referred to the endocrinologist in case of abnormal TSH concentrations. Therefore, we focused on treatment with l-thyroxine or antithyroid agents as a measure of thyroid dysfunction. It must be noted that the large proportion of patients taking l-thyroxine in the present sample (one-third) may be due to the over-surveillance bias in patients treated with lithium as well as to the multiple indications for treatment: hypothyroidism (lithium- and/or autoimmunity-related), hormone replacement after thyroidectomy (for goitre or cancer), and TSH-suppressing therapy of nodular goitre. The apparent association between treatment with l-thyroxine and stage G3b (Table 2) vanished in the adjusted analyses (Tables 3 and 4).

At variance with the present study which was based on specific treatments, population-based surveys of renal and thyroid function have focused on clinical or subclinical hypothyroidism (increased TSH concentrations with normal thyroid hormone concentrations). Hypothyroidism or increased TSH have been found associated with lower eGFR or impaired kidney function in several cross-sectional surveys, mostly European (Lo et al. 2005; Chonchol et al. 2008; Targher et al. 2009; Asvold et al. 2011; Ye et al. 2013; Meuwese et al. 2014; Rhee et al. 2015). On the other hand, results from prospective studies have not been consistent. In fact, for example, an American and a Chinese survey reported that elevated TSH were not associated with the development of incident CKD during follow-up (Schultheiss et al. 2016; Huang et al. 2016), a large South Korean study reported that high levels of TSH were associated with an increased risk of incident CKD (Zhang et al. 2014), whereas the Rotterdam Study reported that higher TSH levels were associated with lower CKD incidence (Chaker et al. 2016).

### Thyroid antibodies

With regard to the role of thyroid antibodies, the absence of control groups of either lithium naïve patients or healthy individuals does not allow us to determine whether the long-term exposure to lithium is a necessary condition or thyroid antibodies may be nephrotoxic in themselves.

Thyroid function has been studied in the general population or in clinical samples with various degrees of CKD (for review, see Iglesias et al. 2017), but the inclusion of antibodies in the panel of thyroid markers has been very rare. To our knowledge, there are only two relevant but contrasting studies (Targher et al. 2009; Schultheiss et al. 2016).

Targher et al. (2009) surveyed cross-sectionally the database of a clinical chemistry laboratory in the north of Italy and found an excess of AbTPO and/or AbTG among individuals with an eGFR lower than 60 mL/min/1.73 m^2^ (18/53 = 34%) compared to the remaining individuals (195/862 = 23%). Age- and sex-adjusted *P* value in logistic regression analysis was significant (0.01). Data included 16 participants with an eGFR lower than 45 mL/min/1.73 m^2^. On the contrary, the Atherosclerosis Risk in Communities study, a large prospective study of middle-aged American adults (Schultheiss et al. 2016) found no association between AbTPO and reduced kidney function. In their prospective evaluation over a median follow-up time of 19.6 years, incidence of CKD (eGFR lower than 60 mL/min/1.73 m^2^) did not differ between AbTPO-positive and AbTPO-negative participants (odds ratio 0.79; 95% CI = 0.5–1.25; *P* = 0.32; total *N* of events = 1488) (Schultheiss et al. 2016).

We first suspected that thyroid antibodies may play a role in CKD of patients treated with lithium when we collected the data for our recent collaboration study (Tondo et al. 2017). In fact, we contributed with 30 patients to the study and we were impressed by the high prevalence of circulating thyroid antibodies among the patients who had eventually manifested severe CKD (7/14 = 50%). The present study confirms that circulating thyroid antibodies can contribute to the severe decline of eGFR in patients treated with lithium.

### Mechanisms

We can only speculate about the potential mechanisms involved in the excess of thyroid-antibody-related CKD in our cohort of patients treated with lithium, because no renal biopsy data were available.

Case reports have repeatedly associated nephropathy with thyroid antibodies over the last four decades. In some cases, deposition of immune complexes was documented. For example, Jordan et al. (1978) reported a case with immune complex glomerulonephritis and hypothyroidism. Granular glomerular basement membrane and mesangial staining were detected by indirect immunofluorescence, with antibody specific for thyroglobulin and thyroid microsomal antigen.

From the clinical point of view, the majority of the cases reported to date were nephrotic syndrome rather than reduced eGFR (for review, see Santoro et al. 2017). With regard to the relationship between pathological and clinical findings, the most informative contribution has been provided by Koçak et al. (2012) who reviewed retrospectively 28 patients with Hashimoto’s thyroiditis referred to their department because of unexplained haematuria, proteinuria or renal impairment. They concluded that glomerular pathologies associated with Hashimoto’s thyroiditis are similar to those in the general population. The most common lesions were membranous glomerulonephritis, focal segmental glomerulosclerosis and immunoglobulin A nephritis. Proteinuria and glomerular filtration rate were found independent of thyroid hormone concentrations and thyroid antibodies titres. Of the 28 cases, 12 (43%) had an eGFR lower than 60 mL/min/1.73 m^2^, whereas proteinuria was found in 24 (86%), nephrotic range proteinuria in 11 (39%), and haematuria in 11 (39%).

Data on prevalence of proteinuria and haematuria were not available for the present study.

Besides the potential deposition of immune complexes, other mechanisms have been involved in the various diseases that have been found associated with circulating thyroid antibodies. This does not necessarily mean that thyroid antibodies play a direct role in all the observed cases. As circulating thyroid antibodies are very prevalent in the population, their concomitant occurrence with other prevalent diseases may be a coincidence. For example, a Sardinian survey found an overall prevalence of AbTPO of 174/789 (22%) in women and 30/444 (7%) in men (Loviselli et al. 1999). With regard to the psychiatric presentation of the so-called Hashimoto’s encephalopathy, we have suggested that it may be dependent on a diffuse vasculitis (Bocchetta et al. 2007, 2016). In turn, vasculitis may be due to concurrent antibodies: for example, Yoneda et al. (2007) have suggested that antibodies against the amino terminal region of alpha-enolase play a role in Hashimoto’s encephalopathy. Because alpha-enolase is expressed in vascular endothelial cells, autoantibodies against this enzyme may be associated with vasculitis. Moreover, alpha-enolase has been identified among intrinsic renal antigens that are targets of nephritogenic antibodies. Indeed, in situ formation of immune complexes is a well-recognised mechanism of renal injury, as observed in systemic autoimmune disorders (Migliorini et al. 2002).

On the other hand, vasculitis or cross-reactivity between antigens are among the mechanisms suggested to explain the cases of glomerulopathies associated with autoimmune thyroiditis (Santoro et al. 2017).

## Conclusions

Whatever the mechanism, the potential role of thyroid antibodies in the decline of glomerular filtration in patients with mood disorders is relevant, given their frequent co-occurrence.

Given the current uncertainty regarding the potential role of thyroid antibodies in CKD due to the paucity of studies on the general population and the lack of a control group of patients with mood disorders never treated with lithium in the present study, it is yet to be established whether the long-term exposure to lithium is a necessary condition to allow the manifestation of the effects of thyroid antibodies or thyroid antibodies are nephrotoxic in themselves.

If our finding will be confirmed, it might have some clinical implications when discussing the role of lithium with consultant nephrologists in cases of patients with declining eGFR. We suggest the inclusion of thyroid antibodies in the list of risk factors potentially associated with CKD.

## Abbreviations

CI: confidence interval
CKD: chronic kidney disease
eGFR: estimated glomerular filtration rate
AbTPO: anti-thyroid peroxidase antibodies
AbM: anti-thyroid microsomes antibodies
AbTG: anti-thyroglobulin antibodies
TRAb: anti-thyrotropin receptors
TSH: thyroid stimulating hormone
FT4: free thyroxine
T3: triiodothyronine
FT3: free triiodothyronine
